# Virtual Reality-Based Exercise Therapy for Patients with Chronic Musculoskeletal Pain: A Scoping Review

**DOI:** 10.3390/healthcare11172412

**Published:** 2023-08-28

**Authors:** Paraskevi Bilika, Natalia Karampatsou, Giorgos Stavrakakis, Achilleas Paliouras, Yannis Theodorakis, Nikolaos Strimpakos, Eleni Kapreli

**Affiliations:** 1 Clinical Exercise Physiology and Rehabilitation Research Laboratory, Physiotherapy Department, Faculty of Health Sciences, University of Thessaly, 35100 Lamia, Greecegestavrakakis@uth.gr (G.S.); apaliouras@uth.gr (A.P.); ekapreli@uth.gr (E.K.); 2 Exercise Psychology & Quality of Life Research Laboratory, Department of Physical Education and Sport Science, University of Thessaly, 42100 Trikala, Greece; 3 Health Assessment and Quality of Life Research Laboratory, Physiotherapy Department, Faculty of Health Sciences, University of Thessaly, 35100 Lamia, Greece; 4 Division of Musculoskeletal & Dermatological Sciences, Honorary Research Associate, University of Manchester, Manchester M13 9PL, UK

**Keywords:** virtual reality, exercise, exercise therapy, chronic pain, musculoskeletal pain

## Abstract

This scoping review aimed to identify interventions utilizing virtual-reality-based exercise therapy in patients with chronic musculoskeletal pain. Searches were conducted in four databases using descriptors related to virtual reality, exercise, and chronic musculoskeletal pain. Two reviewers screened the titles and abstracts of the studies to assess eligibility, with a third author resolving any discrepancies. Data were extracted and summarized in a narrative format by three independent raters. Clinical trials were evaluated using the PEDro scale to assess the effectiveness of virtual-reality-based exercise therapy in chronic musculoskeletal pain patients. A total of 162 articles were identified from the databases. After applying the inclusion criteria, nine articles were considered suitable for analysis, including six randomized clinical trials. The selected articles were categorized based on study characteristics, virtual-reality-based exercise therapy interventions (including technologies and equipment used), exercise interventions, outcome measures, and effectiveness. The findings indicate that virtual-reality-based exercise therapy shows promising results in reducing pain, improving disability, enhancing range of motion, and increasing treatment satisfaction in patients with chronic musculoskeletal pain. However, it is not possible to conclude that virtual-reality-based exercise therapy is superior to other treatments due to the limited number of available studies, heterogeneity in application protocols, and varying methodological quality. Further research is needed to draw more definitive conclusions.

## 1. Introduction

Chronic musculoskeletal pain (CMP) is a debilitating health condition affecting up to a third of the population on a worldwide scale [1,2]. The clinical presentation of the individuals experiencing CMP is usually accompanied by a disability, sleep disturbances, physical inactivity, and a spectrum of cognitive and emotional factors, such as fear-avoidant behaviors, pain catastrophizing, anxiety, and depression [3,4,5,6]. The complex interactions of the aforementioned factors perpetuate the persistence of the painful experience affecting dramatically the quality of life of CMP patients, while at the same time, the consequences on healthcare and social burden cannot be ignored [1,7].

Apart from pharmacological interventions, exercise has been proposed as the cornerstone for the management of CMP, even though the underlying mechanisms are not yet fully understood [8]. Different types of exercise, such as aerobic and resistance, have been used with mixed results in painful populations [9]. Two distinct subgroups seem to arise after a bout of exercise in CMP patients: the “post-exercise induced analgesia” group and the group with pain facilitation following an exercise session [9,10]. The response to exercise observed in the first group is in accordance with what is usually seen in healthy individuals; activation of the endogenous systems with consequent release of substances triggering a hypoalgesic response [9,10,11]. In the latter group though, this well-orchestrated system is thought to be dysfunctional [9,10]. Ιt is vital to overcome the potential obstacle of decreased compliance and adherence to an exercise regime that may come along with post-exercise pain facilitation, as this has been recently proposed to be a possible path for pain reconceptualization [8,12]. Thus, the adoption of additional strategies could be crucial for achieving this.

The advance of virtual reality (VR) technology and its application in the field of musculoskeletal rehabilitation is getting increasing attention [13]. Despite not always being superior to control or conventional physiotherapy [13], the inclusion of VR tools in the management of painful conditions seems to have a plethora of advantages, such as enjoyment, better compliance, and enhanced visual and auditory feedback [14,15,16]. Furthermore, apart from distraction, which has been studied extensively as one of the key mechanisms behind the ability of VR to affect pain, there is supporting evidence for the use of VR as a tool for improving the affective aspects of pain, coping strategies, gradual exposure, and embodiment [17,18,19].

Recent reviews examined the use of VR as an adjunct to physical therapy or as standalone care, summarizing samples of both acute and chronic pain states, which makes our understanding more diffuse [13]. Additionally, when examining chronic pain populations, studies usually include patients with different pathologies and mechanisms at the same time, which may have influenced the overall conclusions to some extent [20]. Considering the abovementioned details, the aim of this scoping review was to identify and collect the available data on purely musculoskeletal populations suffering from chronic pain where virtual-reality-based exercise therapy (VRET) was used as part of their management. Except for exploring the equipment utilized and both VR protocol, as well as exercise parameters used, this scoping review aimed to fulfill objectives such as identifying research gaps and opportunities for further investigation in the field while providing recommendations on barriers and limitations that need to be addressed, suggesting future research directions for AVRT based on the findings of the scoping review.

## 2. Materials and Methods

This scoping review was conducted using the Preferred Reporting Items for Systematic Review and Metanalysis (PRISMA) Extension for Scoping Review instrument [21].

### 2.1. Identification of Purpose and Research Questions

First, this review aimed to identify the types of VRET interventions that are being used for CMP management. Furthermore, this study sought to evaluate the effectiveness of VRET in reducing pain and improving functional and psychosocial outcomes in patients with CMP. In addition, it examined the characteristics of patients with CMP who are most likely to benefit from VRET. This objective required understanding which subgroups of patients may benefit most from this type of therapy and identifying any patient-related factors that may impact treatment outcomes. Another objective of this review was to identify the potential barriers and facilitators to implementing VRET in clinical settings for CMP management. By identifying these factors, this review aimed to provide insight into how VRET can be integrated into clinical practice to improve patient outcomes. Finally, this review highlighted gaps in the current literature and identified areas for future research on VRET for CMP management. By doing so, this review aimed to contribute to developing a robust evidence base for using VRET in CMP management.

The research questions concerned four main topics of interest: the technology, and equipment itself, the type of exercise, the targeted patients, and the study’s outcomes and effectiveness of the VRET. Table 1 presents the research questions explored in the scoping review based on our research interest.

### 2.2. Search Strategy

A member of the research team with prior experience in systematic reviews (P.B.) conducted an electronic literature search in four databases, namely, Medline (via PubMed), PEDro, Web of Science, and Scopus, using the following keywords: “virtual reality”, “exercise”, and “chronic musculoskeletal pain”. The final search strategy for MEDLINE can be found in Supplementary Materials (Table S1). Databases were searched from their inception to March 2023. There were no restrictions on the publication date. For the sake of ensuring a comprehensive review, an additional step was taken wherein the reference lists of the included articles were reviewed. This was done with the aim of identifying any relevant articles that might have been missed during the initial database search. By examining the reference lists, we aimed to minimize the chances of overlooking any pertinent studies and to ensure that our review encompassed as much relevant literature as possible.

### 2.3. Eligibility Criteria for Study Selection

To meet the inclusion criteria, the studies involved individuals who were over 18 years old and had experienced CMP. Chronic pain is generally defined as pain that persists for more than three months, beyond the expected healing time of the underlying injury or illness [22]. Additionally, all VRET interventions provided via head-mounted devices or screens were included. Only the studies involving fully immersive VR interventions were included. Studies reporting participant perspectives on characteristics of VRET, guidelines, and systematic reviews with specific recommendations for VRET characteristics were considered eligible. Clinical trials that used VRET as a specific intervention were also considered eligible for this review. While the primary focus of the review was not on clinical outcomes, these trials could report a broad range of clinical outcomes, such as pain severity, pressure pain threshold, physical function, disability, kinesiophobia, anxiety, and catastrophizing.

Studies that were not published in English were excluded. This scoping review did not consider results from abstracts or conference presentations, book reviews, book chapters, narrative reviews, case series, commentaries, letters to the editor, editorials, and protocols. In the case of incomplete data or unavailability of a study online, one reviewer (P.B.) contacted the authors. The authors were given two weeks to respond to our queries. If there was no response within this timeframe, the study was excluded due to the strict timeline of the study.

### 2.4. Screening

Two independent reviewers (N.K. and G.S.) screened titles and abstracts for eligibility. In the cases of disagreement, a third researcher (P.B.) was consulted to resolve them. The process was carried out using the Rayyan platform, available at https://www.rayyan.ai/, accessed on 31 May 2023.

### 2.5. Data Extraction and Results Synthesis

The relevant data were extracted from the selected studies to effectively address the research questions. The data extracted included study design, sample, setting, VR equipment, exercise type, outcomes, facilitators, barriers, and key findings (Table S2). A data-charting form was collaboratively developed by two reviewers (E.K. and P.B.) to determine the variables that needed to be extracted. Following this, three independent reviewers (G.S., N.K. and P.B.) individually charted the data using the agreed-upon form. Subsequently, they engaged in thorough discussions regarding the results and iteratively updated the data-charting form throughout the process.

### 2.6. Methodological Quality of RCTs

To assess the effectiveness of VRET, as a secondary purpose of this review, the included clinical trials were fully reviewed and scored by two independent researchers using the PEDro scale [23]. Some authors and researchers suggested general interpretations for the total PEDro scores based on their clinical experience. Scores from 0 to 3 are considered “poor”, scores of 4 to 5 are labeled as “fair”, scores ranging from 6 to 8 are considered “good”, and scores of 9 to 10 are considered “excellent” [24]. These classifications can provide a rough indication of study quality, but they should be interpreted with caution as they are not universally agreed upon or formally validated. It is worth mentioning that the interpretation of study quality should not solely rely on the total PEDro score but should also consider the individual PEDro items and other relevant factors when assessing the reliability and applicability of the research findings.

## 3. Results

### 3.1. Literature Search

**Figure 1:**
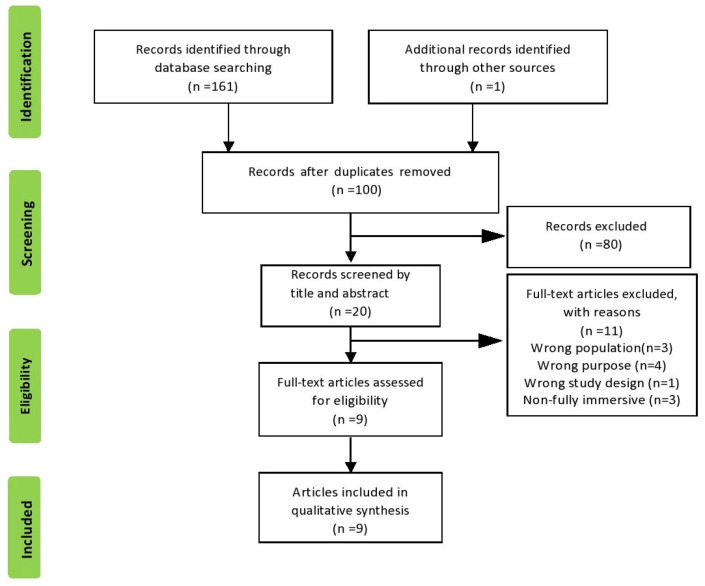
PRISMA flow diagram describing the article selection process.

A total of 161 articles were initially retrieved from various databases, including 40 from PubMed, 8 from PEDro, 79 from Scopus, 34 from Web of Science, and 1 from other sources (Figure 1). To ensure accuracy and eliminate redundancy, duplicate articles were removed, resulting in a reduced set of 100 unique studies.

Subsequently, a screening process was conducted based on the titles and abstracts of these articles, resulting in a selection of 20 articles that met the initial inclusion and exclusion criteria. However, upon further scrutiny of the full texts of the 20 selected articles, 11 articles were excluded as they either involved populations different from the target population or had different purposes, were focused on non-immersive VR, or were cross-sectional studies. Upon reviewing the reference lists of the included articles, we did not retrieve any additional relevant papers that necessitated inclusion in the current scoping review. Finally, nine studies were included in the qualitative synthesis.

### 3.2. Study Characteristics

In this review, a total of nine studies were included that reported on clinical trials utilizing VRET. Table S2 provides a comprehensive table summarizing the general characteristics of all the clinical studies included. Among the included studies, the majority (five out of nine) of the interventions were conducted in university settings. One study was conducted in a hospital; one in a health center; and two interventions were home-based, meaning they were conducted in participants’ own homes. Furthermore, one was classified as a pilot study [25] and one as an open pilot and feasibility study [26]. The remaining studies were categorized as randomized controlled trials, providing a more robust comparison between intervention and control groups [27,28,29,30,31,32].

**Figure 2:**
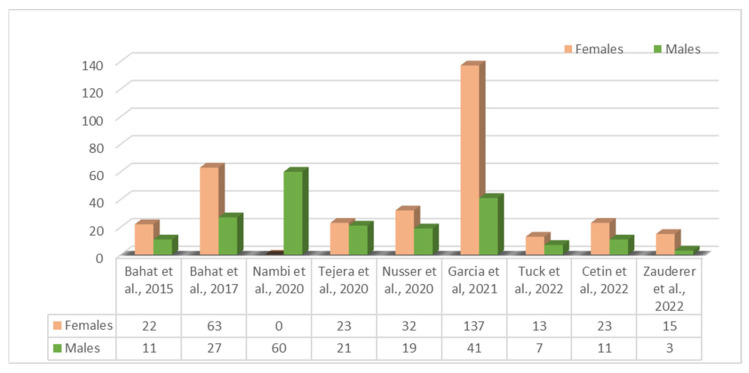
Number of patients categorized by gender per study [25,26,27,28,29,30,31,32,33].

The studies that met the eligibility criteria included a total of 528 chronic pain patients. Out of these, six studies specifically focused on chronic neck pain patients [26,28,29,30,31,33]; two studies targeted low back pain [27,32]; and one study included a broader population of patients with primary musculoskeletal pain, including chest, hip, knee, leg, foot, and pelvis pain [25]. The minimum sample size of patients was 18 and the maximum sample size was 178. Figure 2 displays the distribution of male and female patients across the included studies.

### 3.3. Virtual-Reality-Based Exercise Treatment

**Figure 3:**
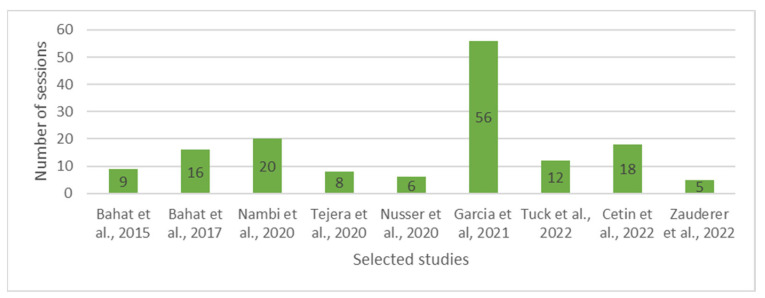
Total number of intervention sessions in the VR group per study [25,26,27,28,29,30,31,32,33].

In all of the included studies, the requirement for VR technology was that it had to be fully immersive. This means that participants were fully engrossed in a virtual environment through visual and auditory stimulation. The hardware utilized for achieving this immersive experience varied across the studies and can be found in the Supplementary Materials (Table S2). However, it is worth noting that in all nine studies, a head-mounted display (HMD) was used as part of the VR system. The HMD provided a virtual visual experience by presenting images or videos directly to the participant’s eyes, which enhanced the sense of immersion and presence within the virtual environment. The games in the included studies exhibited a range of designs, with some being relatively simple, while others featured more complex mechanics. For instance, some studies incorporated games with basic designs, such as shooting activities, during the VR experience [32]. On the other hand, more complex game mechanics were employed in certain studies, including games like “Doritos VR Battle” and “Brick Buster VR” [25,26]. These games likely offered more engaging and interactive experiences, potentially enhancing the immersive nature of the VR intervention and providing additional elements of enjoyment and entertainment for the participants. Out of the nine studies included in the review, the majority (six out of nine) reported having physiotherapists present to supervise the VR sessions [25,26,28,29,30,31]. The total count of intervention sessions varied across the studies and encompassed a range spanning from 5 to 56 sessions (Figure 3).

The studies included in the review combined VR with various types of exercises, such as home kinetic training [30]; sensorimotor training [28,33]; core stability exercises [32]; neck movements throughout the full range of motion [31]; and exercise therapy that incorporated aerobic, mobility, and muscle strengthening exercises [26]. Across all the included studies, the exercise interventions targeted specific body areas, such as the neck and back. However, some studies did not provide specific details about certain parameters of the exercises (Table S2). For a comprehensive overview of the exercise protocols used in the intervention groups of each study, please refer to the Supplementary Materials (Table S2), which provides detailed information.

### 3.4. Outcomes Measures

All studies had baseline and end-line assessments, and five of them had follow-up assessments in 3 months [26,29,30,31] or 6 months [32]. In total, 35 outcome measures were identified. These measures were categorized into four main categories for analysis: pain-related measures; functional measures; psychosocial measures; and other measures that encompassed factors such as satisfaction, feasibility, usability, and acceptability. Across all the studies, pain intensity was evaluated using various standardized measures. The most commonly used pain intensity assessments were the Visual Analogue Scale (VAS) in six out of nine studies and the Numerical Rating Scale (NRS) in two out of nine studies. Additionally, one study employed the Brief Pain Inventory (BPI) and another study utilized the Defense and Veterans Pain Rating Scale (DVPRS-11) to assess pain intensity. Furthermore, other pain-related measures that were reported in the studies included assessments of continued pain modulation, temporal summation, pressure pain thresholds, and the presence of dizziness and headaches (Table S2). Among the functional measures employed in the studies, the active range of motion (AROM) was the most commonly used measure, being utilized in six out of the nine studies. Additionally, the Neck Disability Index (NDI) was employed in four out of the nine studies to assess functional outcomes. For the assessment of psychosocial measures, the Tampa Scale of Kinesiophobia (TSK) (six out of nine) and the Pain Catastrophizing Scale (PCS) (two out of nine studies) were the more commonly utilized measures (Table S2). The inclusion of measuring blood serum stress hormones as an objective psychological marker in one of the studies is an interesting approach [32]. This objective measurement adds an additional layer of scientific rigor and objective assessment to the evaluation of the psychological effects of the interventions on participants. Table S2 details the outcome measures for each study.

### 3.5. Effectiveness of VRET

To evaluate the effectiveness of VRET in patients with CMP, only randomized clinical trials from the selected studies were included. Consequently, the search was narrowed down to six relevant studies [27,28,29,30,31,32]. These studies underwent quality assessment using the PEDro scale, which is commonly used to evaluate the methodological quality of randomized clinical trials. The results of the quality assessment, along with the main findings of each study, are summarized in Table S3. This table provides an overview of the quality of the included studies and highlights the key findings of each randomized clinical trial regarding the effectiveness of VRET in the management of CMP.

**Figure 4:**
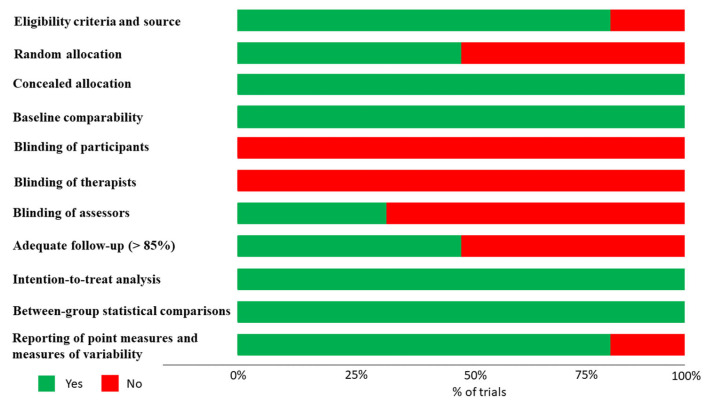
Methodological quality of RCTs.

Based on the quality assessment, one study demonstrated fair methodological quality with a score of 5 out of 10 [28]. Five studies were rated as having good methodological quality, ranging from 6 to 7 out of 10 [27,29,30,31,33]. The criteria that were most commonly not met across the studies were related to blinding of the therapists, assessors, and subjects, as well as the attainment of measures from over 85% of the initially allocated subjects’ groups for at least one key outcome due to dropout rates (Figure 4 and Table S3).

### 3.6. Pain Intensity

In general, pain intensity was found to be reduced across all the studies. However, it is worth noting that in one particular study, there was no significant or substantial reduction in pain intensity observed [28]. Τhis particular study focused on patients with chronic neck pain presented a fair methodological quality and had a small sample size.

Out of the included studies, only two of them reported findings suggesting that VRAE was superior in pain intensity reduction relative to other treatments [27,30]. In the study by Garcia et al. [27], the VRAE intervention called EaseVRx, which incorporated pain education, relaxation exercises, mindful escape (360 videos), pain distraction games, and dynamic breathing exercises, was found to be superior to a non-immersive sham VR intervention in patients with chronic low back pain. Additionally, in the study by Bahat et al. [30], kinematic training using VR was found to have a better improvement in pain intensity compared with kinematic training using a laser in patients with chronic neck pain. Both studies had a good methodological quality based on the PEDro scale. On the other hand, one study, also with good methodological quality, showed that VR interventions did not demonstrate superiority over the control group in terms of pain reduction [31].

### 3.7. Disability

In three randomized clinical trials [29,30,31], the Neck Disability Index (NDI) was utilized to assess disability in patients with chronic neck pain. Within-group analyses consistently demonstrated reductions in NDI scores in the VR group across all three studies immediately post-intervention [29,30] and at the 3-month follow-up [31]. However, when considering the studies that included a control group, only two out of three [30,31] found a significant difference in NDI scores between the VR group and the control group. In contrast, Bahat et al. [29] reported that the VR intervention combined with kinetic training did not show superiority over the kinetic training group without VR in terms of reducing disability, as indicated by the NDI scores.

### 3.8. ROM

Active range of motion (AROM) was evaluated exclusively in patients with chronic neck pain across four randomized clinical trials [28,29,30,31]. Out of these four studies, two reported a significant increase in the AROM in the VR group [28,29]. However, only one study found a significant difference in the within-group analyses [31]. For detailed information about the specific movements that were influenced by the VR interventions, please refer to Table S3. The table provides additional insights into the specific range of motion improvements observed in each study, highlighting the movements that were positively impacted by the VR interventions.

### 3.9. Kinesiophobia

The TSK was utilized to assess kinesiophobia in both pre- and post-intervention assessments across the selected randomized clinical trials. Among these studies, TSK was included in the assessment protocol of four studies. Out of these four studies, two reported a significant reduction in TSK scores within the VR group [31,32] based on within-group analyses. Conversely, the other two studies did not find a significant difference between the pre- and post-intervention TSK assessments [29,30].

In the study conducted by Tejera et al. [31], kinesiophobia was significantly more reduced in the VR group compared with the control group at the 3-month follow-up assessment. In both the studies conducted by Sarig Bahat et al. [29] and Sarig Bahat et al. [30] no significant differences were found between groups in terms of kinesiophobia.

### 3.10. Pain Catastrophizing

Tejera et al. [31] assessed the pain catastrophizing in patients with chronic neck pain. Despite a reduction in pain catastrophizing in the VR group, no significant difference between the VR and the control group was observed. Similar results were published by Garcia et al. [27] in their study assessing patients with chronic low back pain.

### 3.11. Pressure Pain Threshold

A significant increase in PPTs in the right and left articular pillars of C1/C2 and C5/C6 was observed in the VR group compared with the motor control group in patients with chronic neck pain [28]. No significant differences in PPTs in the tibialis anterior and upper trapezius were found between the different groups (VR vs. control group and VR vs. motor control group) [28,31].

### 3.12. Temporal Summation and Conditioned Pain Modulation

Among the included studies, only one study incorporated objective measures related to central sensitization, including CPM and TS [31]. In the analysis that compared the VR group with the control group, no significant difference was observed for CPM. However, for TS, significant improvement was found in the VR group compared with the control group at the 1-month follow-up assessment [31].

### 3.13. Blood Serum Hormones

The assessment of blood serum stress hormones revealed that subjects with chronic low back pain exhibited a slightly greater improvement when using VR compared with isokinetic training [32]. This finding suggests that the VR intervention had a positive effect on stress hormone levels in individuals with chronic low back pain, indicating a potential stress reduction or modulation through the use of VR.

### 3.14. Satisfaction

The findings indicate that VR interventions showed significant effect sizes favoring VR over the waitlist control group in terms of treatment satisfaction and perceived improvement. These effect sizes indicate large and substantial differences in treatment satisfaction and perceived improvement between the VR intervention and the waitlist control group [25]. Additionally, the effect sizes also suggest small but still statistically significant differences favoring VR over treatment as usual (TAU) [25] and a 2D VR intervention [27] in terms of treatment satisfaction and perceived improvement. Although the effect sizes for the comparison between VR and TAU are smaller than those for VR versus the waitlist, they still indicate meaningful differences favoring VR in terms of treatment satisfaction and perceived improvement. The large effect sizes in the comparison with the waitlist control group and the small but statistically significant effect sizes in the comparison with TAU suggest that VR interventions have the potential to enhance patient satisfaction and perceived improvement in clinical settings. In general, good satisfaction was observed in all studies [25,26,27,29].

### 3.15. Qualitative Analyses

In addition to collecting quantitative data, two recent studies included qualitative analyses to gain deeper insights from participants regarding the applicability, acceptance, and satisfaction of using VR.

Tuck et al. [25] conducted semistructured interviews with the first seven participants who completed the eight VR treatment sessions. The interviews aimed to assess the participants’ expectations, perceived usefulness of the VR intervention, changes in pain and function, and suggestions for improvement. The interviews were transcribed and analyzed to identify key themes. According to the results, participants expressed enthusiasm for trying VR rehabilitation and had positive expectations of the treatment. They described VR as more enjoyable, accessible, and achievable compared with their previous experiences with physiotherapy. VR was seen as fun, exciting, and capable of improving activity levels by distracting them from pain. Some participants believed that participating in VR treatment changed the way their nervous system processed pain. However, there was uncertainty among some participants about whether VR could be considered a legitimate treatment, as its enjoyable nature did not align with their expectations of physiotherapy. Moreover, participants reported experiencing pain relief during VR sessions, although this relief was not sustained after treatment. Despite ongoing pain, participants reported increased daily physical activity, improved strength, reduced stiffness, improved sitting and standing tolerances, and greater confidence in engaging in activities of daily living. VR sessions were also described as having a positive impact on participants’ moods, making them feel happier and more content, and providing relief from daily stressors. However, participants whose first language was not English encountered difficulties in understanding the game instructions. Participants emphasized the importance of a comfortable and adjustable headset and the physical space in which the VR was used. The role of a trained physiotherapist in delivering the VR intervention was also discussed, with participants highlighting the importance of having someone with whom they could form a therapeutic alliance.

In accordance with the aforementioned results, participants in the study conducted by Zauderer et al. [26] provided written comments on their experiences with immersive virtual reality (IVR) exercise therapy. The acceptability of the program, including both IVR and non-IVR exercises, was assessed and participant satisfaction was measured. The results showed that the program was well-received, with a mean acceptability score of 75.5/100 for participants and 84.2/100 for physiotherapists. Participant satisfaction was also high, with a mean score of 79.9/100. The comments from participants were categorized into four themes: fun, concentration, safety, and efficacy. Participants found the IVR exercise therapy enjoyable, engaging, and mentally stimulating. They also felt safe during the sessions and perceived the program as effective at addressing their needs. The physiotherapists agreed with these observations and found the rehabilitation program to be appropriate for treating non-specific chronic neck pain.

### 3.16. Barriers

In the selected studies, barriers were reported that underscore some common challenges associated with VR implementation, such as motion sickness, discomfort, and concerns about the weight and practical aspects of the VR equipment. Table 2 provides a comprehensive overview of the barriers reported in the selected studies regarding the use of VR technology.

Dropouts in one study [25] were attributed to comorbid mental health problems, challenges in maintaining regular appointments, and loss of contact following the SARS-CoV-2 lockdown. These factors suggest that the attrition observed in the study was not exclusively tied to the VR treatment itself. There were no adverse effects reported in the remaining groups.

## 4. Discussion

### 4.1. Main Findings

In this scoping review, the aim was to examine the utilization and efficacy of VR in combination with exercise for managing chronic musculoskeletal conditions compared with conventional interventions. Initially, 162 articles were identified from various databases. However, after applying the inclusion criteria defined for this review, only nine articles were deemed suitable for analysis, as described in the Methods section. Out of these, six were randomized clinical trials. The selected articles were assessed and categorized based on five main aspects: study characteristics, VRET interventions (including technologies and equipment utilized), exercise interventions, outcome measures, and effectiveness.

The effectiveness of VRET was evaluated in patients with CMP through a review of seven randomized clinical trials. The studies underwent quality assessment using the PEDro scale, and the results are summarized in Table S2, providing an overview of the study quality and key findings. Overall, pain intensity was reduced in most studies, although one study focusing on chronic low back pain did not show a significant reduction. Two studies suggested that VRET was superior to other treatments in reducing pain intensity. Active range of motion (AROM) improvements were observed in three out of five studies focusing on chronic neck pain. Disability, as assessed using the Neck Disability Index, showed reductions in the VR group across studies, with two studies demonstrating significant differences compared with the control groups. Kinesiophobia, as measured using the TSK, showed mixed results, with two studies reporting significant reductions within the VR group. Pain catastrophizing did not show significant differences between the VR and control groups. PPTs increased significantly in the VR group compared with the control group for specific areas of the neck. Objective measures related to central sensitization showed significant improvement in TS but not CPM in the VR group compared with the control group. Blood serum stress hormone levels were slightly improved with VR compared with isokinetic training in individuals with chronic low back pain. Treatment satisfaction and perceived improvement were significantly higher in the VR group compared with the waitlist control group and the treatment-as-usual (TAU) group. These conflicting findings highlight the need for further research to better understand the circumstances in which VR interventions may be most effective in pain reduction and to identify potential factors that influence treatment outcomes. Continued investigation and replication of studies in this area will contribute to a more comprehensive understanding of the effectiveness of VR interventions for pain management.

Overall, the findings suggest that VRET can effectively reduce pain, improve disability, enhance range of motion, and increase treatment satisfaction in CMP patients. However, it cannot be concluded that AVTR is superior to other treatments. The limited number of studies available, the heterogeneity in the application protocols, and the varying methodological quality of the studies prevent drawing definitive conclusions.

The design of VR studies poses a significant challenge. Many studies were statistically underpowered, despite claiming positive results. Establishing the clinical efficacy of VR therapy requires large-scale, high-quality randomized controlled trials [34]. Comparative studies should include a suitable control environment to differentiate the effects of VR from the media used. However, few studies adequately addressed this distinction, potentially leading to a failure to determine whether it is the VR experience itself or the specific medium that elicited an effect [35]. Addressing these design issues requires larger-scale, high-quality clinical VR studies to avoid confirmation bias and provide more robust evidence [36].

The studies included in this review utilized a range of hardware and software technologies and interventional sessions, which may introduce variability in the results. Quantifying the volume of exercise or physical activity becomes challenging when different studies employ various games or interventions. The variation in games and interventions across studies makes it difficult to directly compare and measure the exact amount of exercise or physical activity performed in each study. Currently, there are no established specific recommendations for the utilization of active VR in the rehabilitation of patients with CMP. The lack of standardized guidelines or protocols highlights the need for further research and evidence to determine the optimal approaches, parameters, and protocols for incorporating active VR into the rehabilitation of individuals with CPM.

### 4.2. Challenges in the Utilization of VRET

The current findings indicate that there is a limited number of studies that have utilized fully immersive VR in the rehabilitation of patients with CMP. The preference for non-immersive VR systems over fully immersive ones can be attributed to several factors. Non-immersive systems are often more convenient and easier to use, as they do not require specialized glasses or headsets with wires. They are also relatively less expensive and more readily available compared with fully immersive VR systems. These factors contribute to the popularity and widespread adoption of non-immersive VR systems in various applications. The current estimated cost of a complete system required to facilitate a high-quality VR clinical experience is approximately USD 2500 per unit, excluding maintenance expenses [37]. However, the studies that were conducted suggest that the heightened immersion provided by fully immersive VR can have positive effects on patient engagement, motivation, and the feeling of being present in therapeutic activities [38]. This enhanced sense of immersion can contribute to a more engaging and effective rehabilitation experience for patients [36].

On the other hand, the utilization of fully immersive VR in rehabilitation comes with its own set of challenges and barriers [37]. Cybersickness is a persistent challenge in HMD-VR design considerations. Cybersickness refers to a range of symptoms that individuals may experience when using VR or immersive technology. The symptoms of cybersickness can vary between individuals but commonly include nausea, dizziness, disorientation, headache, sweating, fatigue, and general discomfort [39]. These symptoms may arise when there is a mismatch between the visual cues presented in the virtual environment and the sensory input received from the body’s own balance and spatial orientation systems. Cybersickness can impact the user experience, limit the duration of VR sessions, and affect the effectiveness of therapeutic interventions.

A small percentage of patients in the reviewed studies reported experiencing cybersickness [27,29,30]. The literature suggests that the decrease in these percentages can be attributed to the advancements in VR technology over time [40]. To mitigate cybersickness in VR experiences, the literature recommends several strategies. One approach is to reduce sudden changes in the field of view that are not controlled by the player [36]. These unexpected shifts can contribute to discomfort and disorientation. By maintaining a consistent and stable visual perspective, the risk of cybersickness can be minimized. Designing VR experiences with a fixed forward view can also help to alleviate cybersickness. By focusing the player’s view primarily in a fixed forward direction, unnecessary head movements and excessive rotation can be minimized. This stability in visual orientation can reduce the potential for discomfort and enhance the overall comfort of the user [34]. It is important to consider that attitudes and tolerance toward cybersickness may differ among populations less familiar with video games or clinical populations. These populations may be more susceptible to cybersickness or may have additional issues that could exacerbate their discomfort.

Another challenge associated with using HMD VR systems is the weight of the helmet. Heavy or uncomfortable HMDs can contribute to discomfort and decrease overall user satisfaction. In this review, two studies highlighted helmet weight as a common complaint [26,33]. To address this issue, lighter and well-fitted HMD designs can help to alleviate discomfort and improve user experience.

Additionally, another challenge is the discomfort of wearing corrective glasses inside a VR headset, which can be problematic for some users [26]. However, many VR headsets feature adjustable lens spacing mechanisms that allow users to customize the distance between the lenses. This adjustment provides more room for wearing glasses and helps minimize discomfort. By properly adjusting the lens spacing, users can achieve a more comfortable fit and enjoy their VR experience without the added discomfort of wearing glasses.

These reported barriers highlight important considerations for the use of VR technology in clinical settings, emphasizing the need to address factors that can impact user experience and acceptability.

### 4.3. Future Directions

Although there is currently a lack of appropriate protocols for utilizing active VR in the rehabilitation of patients with CPM, standardization efforts and conducting more high-quality studies can provide valuable insights into the underlying mechanisms and effectiveness of VR on physiological, psychological, and functional factors. Nevertheless, it is evident that VR has a positive impact on patients with chronic pain, which contributes to improved compliance with rehabilitation programs and ultimately facilitates behavior change, which is the primary goal of treatment. Looking ahead, VR holds promise as a tool that can be effectively utilized in remote patient rehabilitation.

Due to the diverse nature of patients with CMP, it is crucial to adopt individualized and patient-centered interventions in their approach. Future studies should adopt a comprehensive approach that accounts for the unique requirements of individuals with hypersensitivity, a high level of disability, kinesiophobia, and different pain phenotypes. Incorporating graded activity exposure techniques, pain neuroscience education elements, and personalized facilitators can pave the way for more effective and targeted interventions that cater to the diverse needs of these individuals. This individualized approach will help optimize treatment outcomes and enhance patient satisfaction and engagement in the rehabilitation process. It is crucial to investigate how these technologies can support and enhance traditional therapies while also identifying any potential conflicts or challenges that may arise.

### 4.4. Strengths and Limitations

This study has several strengths and limitations. One of the strengths of this study lies in the inclusion of data from four databases and the examination of reference lists, ensuring the inclusion of relevant studies. Additionally, the assessment of methodological quality and data extraction were conducted by three independent examiners, enhancing the reliability of the findings. However, there are some limitations that should be acknowledged. First, the number of trials included in this review was small, which may limit the generalizability of the findings. Second, there are reservations regarding the adequacy and exhaustiveness of the search strategy, potentially creating space for studies that could have been omitted from the review. Third, the studies in this review had relatively small sample sizes, indicating the need for larger-scale trials to provide more robust evidence. Specifically, there were only two studies that assessed the effects of VR on chronic low back pain, six studies that focused on chronic neck pain, and one study that addressed primary chronic musculoskeletal pain. Lastly, the inclusion of studies only in English may have introduced a language bias, where considering studies in non-English languages could have provided additional insights.

## 5. Conclusions

In conclusion, this scoping review explored the utilization and efficacy of VRET in combination with exercise for managing chronic musculoskeletal conditions. The review included nine articles, six of which were randomized clinical trials. The findings suggest that VRET interventions can effectively reduce pain, improve disability, enhance range of motion, and increase treatment satisfaction in patients with chronic musculoskeletal pain. However, due to the limited number of studies, heterogeneity in application protocols, and varying methodological quality, no definitive conclusions can be drawn regarding the superiority of VRET compared with other treatments.

The review identified several challenges and barriers in the utilization of VRET. These include the discomfort caused by the weight of the headset, cybersickness, and issues related to wearing corrective glasses inside the VR headset.

The scoping review highlights the importance of future research and the need for large-scale, high-quality clinical studies to establish the clinical efficacy of AVR interventions. Overall, VR shows promise as a valuable tool in patient rehabilitation, and continued research will contribute to a better understanding of its effectiveness, optimal protocols, and potential benefits for patients with chronic musculoskeletal pain.

## Figures and Tables

**Table 1:** Research questions.

Q1:	What is the current state of research on the use of VR equipment (e.g., headsets, haptic devices) combined with specific types of therapeutic exercise (e.g., aerobic, anaerobic, motor control exercises) for patients with CMP?
Q2:	What specific parameters of exercise (e.g., frequency, intensity, type, time) are utilized?
Q3:	Do the VR interventions incorporate any motivational or target-setting materials? Do they include any cognitive tasks (dual tasks, memory tasks, divided attention activities)?
Q4:	What are the outcomes that have been measured in studies using VRET for patients with CPM? Which pathologies were treated?
Q5:	What are the benefits and limitations of using VR technology combined with specific types of therapeutic exercise for patients with CMP, and are there any reported adverse effects?
Q6:	What gaps in the literature exist regarding the use of VR technology combined with specific types of therapeutic exercise for patients with CMP, and how could future research address these gaps?

**Table 2:** Reported barriers to the use of VR.

Study	Reported Barriers
Bahat et al., 2015 [29]	4/32 participants experienced motion sickness with the use of the VR device during the assessment.
Bahat et al., 2017 [30]	Out of 14 dropouts at post-intervention assessment, 5 were due to VR-associated sickness and headache.
Nusser et al., 2020 [33]	Complaints about the weight of the helmet and the treatment planning.
Garcia et al., 2021 [27]	7/72 (9.7%) from the VR group reported experiencing nausea and motion sickness during the treatment phase of the study.
Zauderer et al., 2022 [26]	Concerns for both participants and physiotherapists were the weight of the headset, the discomfort of wearing corrective glasses inside the VR headset, and difficulty scoring the pain intensity when wearing the VR device.

## Data Availability

Not applicable.

## Supplementary Materials

The following supporting information can be downloaded from https://www.mdpi.com/article/10.3390/healthcare11172412/s1: Table S1. Search strategy. Table S2. Characteristics of eligible studies. Table S3. Assessment of methodological quality based on PEDro scale and main findings per study.
