# Supplementary material for: Virtual Reality-Based Exercise Therapy for Patients with Chronic Musculoskeletal Pain: A Scoping Review
Source: Healthcare (Basel). 2023 Aug 28;11(17):2412. doi: 10.3390/healthcare11172412 (PMC10487144; doi:10.3390/healthcare11172412)
Supplement: Supplementary file 1 [file healthcare-11-02412-s001.zip › healthcare-2556776-supplementary/Table S1. Search strategy.pdf]

Table S1. Search strategy.

**PubMed Search Formula vía NLM. Results:40**
(((("virtual reality"[Text Word] OR "immersion virtual reality"[Text Word] OR "head-mounted display"[Text Word])) AND (exercise[Text Word] OR "physical activity"[Text Word] OR sports[Text Word] OR step*[Text Word] OR bike[Text Word] OR treadmill[Text Word])) AND (psychology[Text Word] OR "mental health"[Text Word] OR "physiological outcomes"[Text Word] OR "psychological outcomes"[Text Word] OR rehabilitation[Text Word] OR therapy [Text Word] OR "rehabilitative process"[Text Word] OR pain[Text Word])) AND ("chronic pain"[Text Word] OR "chronic musculoskeletal pain"[Text Word])

Scopus Search Formula vía ELSEVIER. Results:79

TITLE-ABS-KEY-AUTH ("virtual reality" OR "immersion virtual reality" OR "head-mounted display") AND (exercise OR "physical activity" OR sports OR step* OR bike OR treadmill) AND TITLE-ABS-KEY-AUTH (psychology OR "mental health" OR "physiological outcomes" OR rehabilitation OR therapy OR "rehabilitative process" OR pain) AND TITLE-ABS-KEY-AUTH ("chronic pain" OR "chronic musculoskeletal pain")

Wos Core Collection Searh Formula vía ELSEVIER. Results:34

#1 TS= ("virtual reality" OR "immersion virtual reality" OR “head-mounted display”)
#2 TS= (exercise OR "physical activity" OR sports OR bike OR step* OR treadmill)
#3 TS= (psychology OR "mental health" OR "physiological outcomes" OR rehabilitation OR therapy OR "rehabilitative process" OR pain)
#4 TS= ("chronic pain" OR "chronic musculoskeletal pain")
#5 #1 AND #2 AND #3

PEDro Search Formula. Results: 8

#1
Abstract and Title: virtual reality
Body part: no appropriate value in this field
Subdiscipline: musculoskeletal
Topic: chronic pain
Method: clinical trial
Match all search terms (AND)
Results: 8

#2
Abstract and Title: immersion virtual reality
Body part: no appropriate value in this field
Subdiscipline: musculoskeletal
Topic: chronic pain
Method: clinical trial
Match all search terms (AND)

Results: 0

#3
Abstract and Title: head-mounted display
Body part: no appropriate value in this field
Subdiscipline: musculoskeletal
Topic: chronic pain
Method: clinical trial
Match all search terms (AND)
Results: 0
