# Supplementary material for: Virtual Reality-Based Exercise Therapy for Patients with Chronic Musculoskeletal Pain: A Scoping Review
Source: Healthcare (Basel). 2023 Aug 28;11(17):2412. doi: 10.3390/healthcare11172412 (PMC10487144; doi:10.3390/healthcare11172412)
Supplement: Supplementary file 1 [file healthcare-11-02412-s001.zip › healthcare-2556776-supplementary/Table S2. Characteristics of eligible studies _2.pdf]

Table S2. Characteristics of eligible studies.

**Study**<br>**a. Authors, Year**<br>**b. Study type**<br>**c. Setting (clinic, hospital, rehabilitation center, university, home-based program)**<br>**d. Country**	**Participants**<br>**a. Total number of participants with males' and females' discrimination**<br>**b. Groups discrimination (affected body area) Diagnosis**<br>**c. Average age/ range**	**VR**<br>**a. Hardware**<br>**b. Software**<br>**c. Cognitive tasks (dual tasks, memory tasks, or divided attention activities)**	**Exercise with VR**<br>**a. FITT**<br>**b. Body area(s) involved in VRET**<br>**c. Equipment**<br>**d. Physiotherapist's overview**	**Control Group (CG)/ Other interventions**<br>**a. FITT**<br>**b. Body area(s) involved**<br>**c. Exercise Equipment**<br>**d. Physiotherapist's overview**	**Number of rehabilitation sessions/ frequency**	**Outcomes**<br>**a. Pain Outcomes (tests, measures)**<br>**b. Functional outcomes**<br>**c. Psychosocial outcomes**<br>**d. Satisfaction**<br>**e. Qualitative components**	**Main Findings**
a. Sarig Bahat et al., 2015 [29]<br>b. Randomized controlled study<br>c. University<br>d. Australia	a. 32 (22 females, 11 males)<br>b. chronic neck pain (>3 months and NDI score >10%, idiopathic and traumatic neck pain)<br>c. KTVR 40.63 ± 14.18 (>18 years),<br>KT 41.13 ± 12.59 (>18 years)	a. Head-mounted display with a 3D motion tracker built in (Wrap™ 1200VR by Vuzix, Rochester, New York).<br>b. Unity-pro software, version 3.5(Unity Technologies, San Francisco), Vuzix Software development kit including their calibration and tracking data tools<br>c. Not specified	a. F: at least 3 times/week for 3 weeks and then encouraged to be performed at home training for 30 min, at least three times a week and to continue this following completion of the supervised sessions until the 3-month post-intervention.<br>I: The VR training program was tailored to each participant and progressed according to the patients' performance<br>T: 30 min (15-20min using VR, 10-15min KT in preparation for home exercise<br>T: Movements of the head, Cervical ROM, velocity, static stability of head, head movement accuracy<br>b. Cervical spine<br>c. Not specified<br>d. Supervised Rehabilitation Program	KT group:<br>a. F: 3 times/week for 6 weeks and then encouraged to be performed at-home training for 30 min, at least three times a week, and to continue this following completion of the supervised sessions until the 3-month post-intervention.<br>I: Not specified<br>T: 30 min,<br>T: ROM, fast head movements, head stability, smooth and accurate head movements following a target<br>b. Cervical spine<br>c. Not specified<br>d. yes	3 sessions /week for 3 months	a. Pain Intensity (VAS) NDI, Cervical<br>b. NDI, ROM, and kinematics (VPeak, VMean, TPT%, Static head stability, Head movement accuracy)<br>Static balance, Functional balance(single leg standing task and step test)<br>c. TSK,<br>d. Patient satisfaction GPE (11-point scale (−5 = totally dissatisfied, 0 = no satisfaction,<br>5 = totally satisfied), Dizziness intensity during the last week (0-100mm VAS, home exercise compliance level on a 4-point scale<br>e. –	**Within-group analysis**<br>KTVR: VAS ↓ post-intervention, step test ↑, NDI↓ post-intervention and 3-month follow-up, ROM_{rotL}↑, ROM_{rotR}↑, ROM_{flex}↑ post-intervention and 3-month follow-up, ROM_{Ext}↑ 3-month follow-up, 9/14 velocity module post-intervention, 5/14 velocity module 3-month follow-up, TSK↔, GPE↑ post-intervention, good satisfaction from therapy,<br>KT: NDI↓ post-intervention ROM_{rotL}↑, ROM_{rotR}↑, post-intervention and 3-month follow-up, ROM_{flex}↑3-month follow-up, 2/14 velocity module post-intervention, 9/14 velocity module 3-month follow-up, GPE↑ post-intervention, good satisfaction from therapy, VAS↔, TSK↔, step test ↑<br>**Between-groups analysis**<br>KTVR was not superior to the KT training<br>GPE↑: KTRV>KT 3-month follow-up<br>ROM_{flex}: KTVR >KT post-intervention<br>ROM_{rotR}: KTVR< KT post-intervention<br>Peak velocity (R and L): KTVR< KT 3-month follow-up<br>Mean velocity (R): KTVR< KT 3-month follow-up

a. Sarig Bahat et al., 2018 [30]<br>b. Randomized controlled study<br>c. home-based program<br>d. Saudi Arabia	a. First baseline assessment: 90 participants (27 Male /63 Female), Second assessment 92 participants<br>b. Neck (chronic neck pain)<br>c. VR=48 (38,5) Laser=48 (35,5), CG= 48 (35)	a. Oculus Rift DK1 head-mounted display equipped with 3D motion tracking<br>b. The software was developed using Unity-pro, version 3.5 (Unity Technologies, San Fran). Three modules were developed, including ROM, velocity, and accuracy modules. These modules enable the elicitation of cervical motion by the patient's response to the provided visual stimuli.<br>c. Not specified	a. F: 4 days/week – 4 weeks, I= Not specified, T= 20min (4x5)/day – 20 min per day, T= ROM /velocity/ accuracy<br>b. Neck<br>c. Not specified Minimally Supervised Home Exercise Protocol	Laser group:<br>a. F: 20min day – 4 days/week – 4 weeks, I= Not specified, T= 20 min per day (4x5), T= ROM/accuracy/ velocity<br>b. neck<br>c. a head-mounted laser beam aimed at a 70 by 70 cm poster<br>d. minimally supervised home exercise protocol<br><br>CG:<br>waiting list- no treatment (first allocation)<br>allocate into VR and Laser groups (second assessment)	20min (4x5)/day for 4 days/week for 4 weeks<br>32 sessions for participants from (VR and Laser group from the first allocation), and 16 sessions for participants from the CG who were waiting in the first allocation	a. Pain Intensity (VAS),<br>b. EQ-5D, ROM and kinematics (velocity, TTP%, NVP - Accuracy error, NDI,<br>c. TSK, GPE for changes in neck pain since receiving treatment<br>d. GPE for patient satisfaction with the intervention<br>e. –	**Within-group analysis**<br>VR group: NDI↓, Vmean↑, Vpeak↑, VAS↓, EQ5D↑, TSK↔, NVP↔, ROM↔, TTP%↑ (except TTP_E%), Accuracy error↓ (except accuracy errorE)<br>Laser group: NDI↓, Vmean↑ (except RR), Vpeak↑ (except LR), VAS↓, TSK↔, NVP_E↑, NVP_{RR}↑, ROM↔, TTP%↑ (except TTP_E% & TTP_{RR}%), Accuracy error↔<br>CG: NDI↔, Vmean ↔, Vpeak↔, VAS↔, EQ5D↔, TSK↔, NVP↔, ROM↔, TTP↔, Accuracy error↔<br>**Between-groups analysis**<br>NDI, Vmean, Vpeak (except LR), TTP_F% & TTP_{LR}%, Accuracy error (F & RR): VR>CG<br>VAS, Vmean (except RR), Vpeak (except Flexion and Right Rotation), EQ5D, Accuracy $error_F$: VR> Laser<br>There were no group GPE differences.<br>The number of home exercise sessions was significantly higher for the laser group (mean 18.29 ± 8.63 or 4.5 times per week) compared to the VR group (14.36 ± 5.78 or 3.5 times per week).
a. Nambi et al., 2020 [32]<br>b. Randomized controlled study<br>c. University<br>d. Saudi Arabia	a. 60 males<br>b. Chronic low back pain (≥ 3 months, 4-8/10 pain intensity)/ university students<br>c. VRT 23.2 ± 1.5 years /18-25 years<br>IKT 22.8 ± 1.6 years/ 18-25 years<br>CG 23.3 ± 1.5 years/18-25 years	a. Not specified<br>b. Shooting game/<br>c. The game was executed and controlled by moving the trunk back and forth and left and right according to the signs. The game's level of challenge was determined by factors such as the number of enemies, the angle at which they were thrown, the frequency of shooting, the rate at which enemies flashed, and the number of balls that appeared around the player.	a. F: 5 days/week for 4 weeks, I: it depends on the parameters of the game T: 30min, T: Balance of stability of core muscles<br>b. Trunk<br>c. Not specified<br>d. Not specified	**IKT Group:**<br>a. F: 5 days/week for 4 weeks, I: Not specified T: 15rep/3 sets, between each set 30 seconds rest and between each pace 60 seconds rest, T: flexion-extension of the trunk,<br>b. Trunk<br>c. isokinetic dynamometer<br>d. Not specified<br>**CG:**<br>a. F: 10-15 times/day, I: Not specified, T: Not specified, T: conventional training of the core muscles of	5 sessions /week, 4 weeks	a. Pain Intensity (VAS),<br>b. Blood serum stress hormones<br>c. TSK-17<br>d. –<br>e. –	VR and IKT groups: VAS ↓ after 4 weeks and 6 months, Kinesiophobia ↓ after 4 weeks and 6 months<br>Blood serum stress hormones: subjects with cLBP exhibited a slightly greater improvement when using VR compared to IKT

				the trunk, strength, stretching<br>b. Trunk<br>c. Not specified<br>d. Not specified			
a. Tejera et al., 2020 [31]<br>b. Randomized controlled study<br>c. University<br>d. Spain	a. 44 (21 males, 23 females)<br>b. Non-specific chronic neck pain<br>c. VR Group: 32.72 ± 11.63/ 18-65 years, Control group: 26.68 ± 9.21/ 16-65 years	a. VR Vox Play glasses / Smartphone (LG Q6)<br>b. Full Dive VR, VR Ocean Aquarium 3D<br>c. Participants named the photos and the animals that were displayed. Also, the application presented auditory and sensory stimuli by integrating the sound of the sea.	a. F=2 days/week for 4 weeks, I= Not specified, T= 3 sets of 10 repetitions of each exercise with 30-sec rest between the exercises, T: Cervical neck movements (flex-ext-rotat-tilt-lateral flex) in a sitting position + VR games<br>b. Neck/ cervical spine<br>c. A ball<br>d. supervised rehabilitation program	CG:<br>a. F=2 days/week for 4 weeks, I= Not specified, T= 3 series of 10 repetitions of every exercise, with a 30 s rest between exercises), T= Cervical neck movements (flex-ext-rotat-tilt-lateral flex) in a sitting position<br>b. Neck<br>c. A ball<br>d. supervised rehabilitation program	2 sessions /week for 4 weeks.	a. Pain Intensity (VAS), CPM, TS, PPTs<br>b. AROM, NDI<br>c. PCS, TSK, FABQ, PASS-20<br>d. Not specified<br>e. Not specified	**Within-group analysis**<br>VR: VAS↓, ROM ↔, NDI↓, PPT↑, TSK↓, PCS↓, TS↔, CPM↔, FABQ↓<br>CG: VAS↓, ROM ↔, NDI↓, PPT↑, TSK↓, PCS↓, TS↔, CPM↔, FABQ↓<br>**Between-groups analysis**<br>VAS, ROM, NDI, PCS, PPTs, CPM, TS: VR=Control<br>TSK: VR>Control (VR Group had better improvement in 3 months follow-up)
a. Nusser et al., 2020 [33]<br>b. Randomized controlled pilot study<br>c. University<br>d Ohio	a. 51 (32 females, 19 males)<br>b. non-traumatic chronic neck pain<br>c. CG: 49.8 ± 8.1, VR: 51.2 ± 8.8, SMG: 53.1 ± 5.7	a. modified VR system (Fraunhofer-Institut für Graphische Datenverarbeitung, Darmstadt, Germany), a helmet (Schutzhelm uvex pheos alpine. Fürth, Germany), an integrated monitor (VR head-mounted display 5DT HMD 800-26 2D, League City, Texas, USA).<br>b. Not specified (The subjects were then asked to follow the paths of motion of a virtual planet in this virtual outer space environment.)<br>c. Not specified	a. F: 6 sessions 20 min<br>I: Not specified<br>T: Not specified<br>T: standard rehabilitation program* and 120 min of "neck-specific sensorimotor training" (NSST) using a VR device<br>b. Neck/ cervical spine<br>c. Not specified<br>d. Not specified	CG:<br>F: Not specified<br>I: Not specified<br>T: Not specified<br>T: standard rehabilitation program*,<br>SMG:<br>F: Not specified<br>I: Not specified<br>T: over 4 30-minute group therapy sessions and was instructed by a physiotherapist or a certified sports scientist.<br>T: standard rehabilitation program* + 120 min of "general sensorimotor training". The objective was training and improvement in patients' coordination through skill exercises (e.g. passing an obstacle	3 weeks. Session frequencies are different between groups, and they not specified	a. Pain intensity (neck pain at rest, neck pain during motion,) headache at rest, headache during motion. (NRS)<br>b. ACROM flexion, extension, left rotation, and right rotation, NDI<br>c. –<br>d. –<br>e. –	**With-in group analysis**<br>VRG: neck pain at rest ↓, headaches↓, ACROM↑ in flexion, extension, left rotation, and NDI↓<br><br>**Between-group analysis**<br>headaches (improvement), ACROM in flexion and extension: VRG> CG<br><br>ACROM cervical extension VRG > SMG<br><br>Neck pain during motion: VRG=CG=SMG<br>neck pain at rest: VRG=CG=SMG

				course, dribbling, rope skipping, tossing balls through rings), balance exercises, small<br>e. game forms (e.g. juggling, curling, throwing and catching), and partner games, such as badminton or table tennis.			
a. Garcia et al, 2021 [27]<br>b. Randomized Clinical Trial<br>c. home-based<br>d. USA	a. 179 (41 males, 137 females, 1 other)<br>b. cLBP<br>c. EaseVRx 51.5/18-81 years, Sham VR 51.4/25-81 years	a. Pico G2 4K all-in-one head-mounted VR device<br>b. Pico G2 4K and each VR device included customized software based on each individual's assigned VR treatment group.<br>d. Not specified	EaseVRx group (full immersive):<br>a. F: daily for 56 days, I: Not specified, T = 2-16 minutes in length (average of 6 minutes), T= pain education, relaxation, mindful escape (360 videos), pain distraction games, dynamic breathing.<br>b. Not specified<br>c. Not specified<br>d. Non-supervised rehabilitation program	Sham VR group (non-immersive):<br>a. F= daily for 56 days, I = Not specified T= average duration of sessions closely matching those of EaseVRx, T= viewing nature scenes, not overly relaxing, not aversive, not distracting, not interactive.<br>b. Not specified<br>c. The Sham VR headset displayed 2D nature footage (eg, wildlife in the savannah) with neutral music<br>d. Not specified	Daily for 56 (8 weeks) days with 2-16 minutes in length (average of 6 minutes) = 56 sessions	a. DVPRS-11<br>b. PROMIS,<br>c. PCS, PSEQ-2, CPAQ-8<br>d. SUS<br>e. -	Pain intensity↓, pain-related interference in activity↓, mood↑, and stress post-treatment↓, user satisfaction, patient's Global Impression of Change, PROMIS physical function: EaseVRx > Sham 2D VR<br>Treatment engagement, usability, sleep disturbance: EaseVRx = Sham 2D VR<br>Neither group evidenced a significant change in opioid treatment, pain catastrophizing, pain self-efficacy, and pain acceptance.
a. Tuck et al., 2022 [26]<br>b. Randomized Pilot Study<br>c. hospital-based interdisciplinary chronic pain center,<br>d. New Zealand	a. 20 (13 females, 7 males) VR 10 (8 females, 2 males), Waitlist & TAU 10 (5 females, 5 males)<br>b. chronic primary musculoskeletal pain<br>c. 40.1 ± 16.2/ 18-70 VR 41.3 ±17.7 Waitlist & TAU 38.7±15.3	a. HTC Vive immersive VR system (HTC Corporation)/ head-mounted display accompanying hand sensors and wall-mounted desktop display<br>b. Games that encouraged full-body movements were selected (Fruit Ninja VR, Holodance, Candy Smash VR, QuiVR, NBA 2KVR Experience, Lightblade VR, Bitslap, Space Pirate Trainer, Fancy	a. F: 2 times/week I: not specified T: not specified T: Full-body movements/ physically active tasks<br>b. full body<br>c. Not specified<br>d. Supervised rehabilitation program (4 years of experience in using VR for chronic pain)	Waitlist Group:<br>No exercise/<br>TAU Group:<br>F: Not specified<br>I: Not specified<br>T: 6 weeks<br>T: Pain Neuroscience Education, fear avoidance, and deconditioning, and participants were given home-based exercise regimens and tailored gym-based activity programs focused on	2 sessions /week, 6 weeks	a. BPI<br>b. Daily Activity and step counts were selected by monitors<br>c. TSK, Global impression of change<br>d. Participants indicated their satisfaction with treatment on a scale from 1 (not at all satisfied) to 7 (very satisfied), enjoyment, and	Immersion scores ranged from 8.4 to 9.6, and enjoyment scores ranged from 8.0 to 9.9<br>There were medium ESs favoring VR over the waitlist for a reduction in BPI pain intensity (ES=0.52) and BPI pain interference (ES=0.50).<br>Small ESs favored the waitlist over VR (ES=0.24) and TAU over VR (ES=0.44).<br>None of the groups met the criteria for MCID in kinesiophobia change scores (≥18% reduction)<br>There were small ESs favoring VR over the waitlist for both step count (ES=0.22) and activity scores (ES=0.26). Both VR

		Skiing VR, Doritos VR Battle) and participants were guided to perform physically active tasks within the virtual environment and progressed through VR games at the discretion of the treating physiotherapist<br>c. Not specified		graded activation and exposure therapy.		immersion collected at the end of each VR session<br>e. 7 participants attended 30-60-min semistructured interviews designed to assess their expectations and perceived usefulness of the VR, changes in pain and function, and suggestions for improvement.	and TAU met the criteria for an MCID in daily step counts (≥600-1100 step increase There were very large ESs favoring VR over the waitlist for treatment satisfaction (ES=1.18) and perceived improvement (ES=1.31) and small ESs favoring VR over TAU for treatment satisfaction (ES=0.28) and perceived improvement (ES=0.24<br>Based on the qualitative data, participants in the study expressed enjoyment of the virtual reality (VR) treatment and found it beneficial for their chronic pain rehabilitation. The outcomes of the VR treatment in this pilot study were found to be better than receiving no treatment and appeared to be at least comparable to standard physiotherapy.
a. Cetin et al., 2022 [28]<br>b. Randomized Clinical Trial<br>c. University<br>d. Turkey	a. 34 (23 females/ 11 males)<br>b. chronic neck pain (≥ 6 months and NDI ≥ 20%)<br>c. VR group= 40.0 ± 11.88/ 18-65 years, MC group= 41.94 ± 10.76 /18-65 years	a. Oculus Go VR glasses<br>b. "Ocean Rift" and "Gala 360"<br>c. They were encouraged to move their necks by expressions	a. F: 3 days/week for 6 weeks.<br>I: Not specified,<br>T:20 min VR/20 min motor control exercises (5 repetitions for each exercise),<br>T: were asked to look in all directions during the VR application. (full neck movement)<br>b. Neck<br>c. VR headset<br>d. supervised rehabilitation program	a. MC group:<br>F: 3 days/week for 6 weeks,<br>I: Not specified<br>T:40min,<br>T: strengthening of the deep cervical flexors, deep cervical extensors, and axioscapular muscles, stretching exercises, and postural correction exercises (3-level treatment protocol by Jull).<br>b. neck<br>c. elastic bands and an exercise ball<br>d. supervised rehabilitation program	3 sessions/ week for 6 weeks	a. Pain Intensity (VAS), PPTs<br>b. ROM & JPSE Muscle performance (dynamometry) Endurance (pressure biofeedback device) Symptoms and functional limitations: ProFitMap-Neck, Quality of life: SF-36<br>c. HADS<br>d. –<br>e. –	**Within-group analysis**<br>VR: ROM↑, JPSEs↑, PPTs↑, muscle strength↑, muscle endurance↑, ProFitMap-Neck↓, some parameters of SF-36↑<br>MC: ROM↑ (except lateral flexions), some PPTs↑, muscle strength↑, ProFitMap-Neck↓, some parameters of SF-36↑<br>Between-groups analysis<br>VAS, muscle strength, muscle endurance, HADS, SF-36, ProFitMap-Neck, PPTUT, PPTTA: VR=MC<br>PPTC1/C2 & PPTC5/C6, JPSEs: VR>MC<br>ProFitMap-Neck (functional limitation index) VR>MC
a. Zauderer et al., 2022 [25]<br>b. Open pilot and feasibility study<br>c. health center<br>d. France	a. 18 (15 females, 3 males)<br>b. NsCNP (≥ 3 months and pain intensity ≥ 40/100)<br>c. 52.8 ±14.3/ ≥18	a. IVR device (KineQuantum), HTC VIVE headset, a consumer virtual reality headset with 32 optical sensors<br>b. Software driver: SteamVR<br>c. Not specified	a. F: 5 sessions<br>I: Not specified<br>T: 3 hours/session<br>T: standardized IVR exercise therapy including active cervical spine ROM and eye–neck coordination exercises using the IVR device (18 min) and non-IVR exercise	No CG	5 sessions	a. Pain intensity (NRS 0-100)<br>b. active cervical-spine RoM, NPDS, head repositioning test,<br>c. TSK<br>d. Feasibility (defined as the	Mean participant satisfaction was 79.9 (±15.9)/100<br>The mean acceptability of the program (as a whole, including IVR and non-IVR exercises) was 75.5 (±20.1)/100 for participants (n = 15) and 84.2 (13.6)/100 for physiotherapists (n = 3)<br>After 5 sessions, there were small improvements from baseline in IVR-

			therapy including aerobic, mobility and muscle strengthening exercises, and provision and teaching of a personalized home-based exercise program (4 to 6 exercises/ 2h and 30min)<br>b. full body<br>c. bands<br>d. supervised rehabilitation program			acceptance rate for participation and actual participation rate (in all 5 sessions), percentage of responders to the self-administered questionnaires at baseline (V1), at the end of the 5 sessions (V2), and at the last follow-up (V3); acceptability of the program at V2 (NRS 0-100); and participant satisfaction with the program at V2 (NRS 0-100), Physiotherapist acceptability (NRS 0-100)<br>e. –	measured cervical spine ROM for flexion, mean (SD) 3.9 (14.9) °, and left axial rotation, mean 6.1 (21.7) °. At V3, mean changes from baseline were −17.7 (27.8)/100 for pain intensity and −12.7 (21.5)/100 for neck-specific activity limitations using the NPDS<br>Summary of patient comments on the IVR therapy program:<br>Patients find IVR exercise therapy fun, enjoyable, and pleasant.<br>IVR therapy requires concentration and helps occupy the brain, reducing pain during movement and increasing the range of motion.<br>Participants feel safe during the therapy and less anxious about movement.<br>There is a belief that IVR exercise therapy could be effective in improving neck pain, maintaining neck mobility, and restoring normal neck function.<br>Some patients express a willingness to participate in another rehabilitation program using IVR if it is shown to be beneficial and can prevent pain attacks

AROM= Active Range of Motion, BPI= Brief Pain Inventory, CG= Control Group, cLBP= Chronic Low Back Pain, CPAQ-8=Chronic Pain Acceptance Questionnaire, CPM=Conditioned Pain Modulation, CROM = cervical range of movement, DVPRS-11=Defense and Veterans Pain Rating Scale, EQ-5D = EuroQol 5 (dimension), FABQ=Fear- avoidance beliefs questionnaire, FITT= Frequency-Intensity-Time-Type, GPE= Global perceived effect, IKT=Isokinetic Training, IVR= Immersive Virtual Reality, JPSE= Joint Position Sense Error, KT= Kinematic Training, KTVR=Kinematic Training plus Virtual Reality, MPQ= McGill Pain Questionnaire, NPDS= Neck Pain and Disability Scale, NDI= Neck Disability Index, NRS= Numerical Rating Scale, NsCNP= Non-specific Chronic Neck Pain, NVP = Number of velocity peaks, NVP_E = Number of velocity peaks for Extension, NVP_{RR}= Number of velocity peaks for Right Rotation, PCS= Pain Catastrophizing Scale, PPT = Pressure Pain Threshold, Pro-Fit-Map neck=Profile Fitness Mapping Neck Questionnaire, PROMIS= Physical Function and Sleep Disturbance, PSEQ-2= Pain Self-Efficacy Questionnaire (2-item), ROM= Range of Motion, SMG= Sensorimotor Group, STAI= State-Trait Anxiety Inventory, SUS= System Usability Scale, TS= Temporal Summation, TSK=Tampa Scale for Kinesiophobia, PASS-20=Pain Anxiety Symptoms Scale Short Form, TTP% =Time to peak velocity percentage, TTP_E%= Time to peak velocity percentage for Extension, TTP_F%= Time to peak velocity percentage for Flexion, TTP_{RR}%= Time to peak velocity percentage for Extension for Left Rotation, TTP_{RR}%= Time to peak velocity percentage for Extension for Right Rotation, VAS= Visual Analoge Scale, Vmean-= mean velocity, Vpeak= peak velocity, VRET=Virtual reality-based Exercise Therapy, VRT= Virtual Reality Training, *Standard Rehabilitation Program: consisted of a comprehensive approach to treating patients, which involved a combination of individual and group therapies led by physiotherapists and certified sports scientists. The program included various types of exercise therapy tailored to both general and neck-specific needs, such as strengthening exercises, mobilization techniques, relaxation methods, medical training therapy, functional gymnastics, aqua therapy, physical therapy, and traditional "back school" exercises. The specific adaptation of exercises was determined by the individual therapist based on their clinical expertise. In addition to the exercise therapies, patients also benefited from special lectures delivered by orthopedists and psychologists.
