# Supplementary material for: Virtual Reality-Based Exercise Therapy for Patients with Chronic Musculoskeletal Pain: A Scoping Review
Source: Healthcare (Basel). 2023 Aug 28;11(17):2412. doi: 10.3390/healthcare11172412 (PMC10487144; doi:10.3390/healthcare11172412)
Supplement: Supplementary file 1 [file healthcare-11-02412-s001.zip › healthcare-2556776-supplementary/Table S3. PEDRO & Effectiveness.pdf]

Table S3. Assessment of Methodological Quality Based on PEDro Scale and Main Findings per Study.

Study	Eligibility criteria & source	Random allocation	Concealed allocation	Baseline comparability	Blinding of participants	Blinding of therapists	Blinding of assessors	Adequate follow-up (> 85%)	Intention-to-treat analysis	Between-group statistical comparisons	Reporting of point measures & measures of variability	Total Score	Rating	Main Findings
Sarig Bahat et al., 2015 [29]	**Y**	**Y**	**Y**	**Y**	**N**	**N**	**Y**	**N**	**Y**	**Y**	**Y**	**7/10**	**Good**	**Within-group analysis**<br>KTVR: VAS ↓ post-intervention, step test ↑, NDI↓ post-intervention and 3-month follow-up, ROM_{rotL}↑, ROM_{rotR}↑, ROM_{flex}↑ post-intervention and 3-month follow-up, ROM_{Ext}↑ 3-month follow-up, 9/14 velocity module post-intervention, 5/14 velocity module 3-month follow-up, TSK↔, GPE↑ post-intervention, good satisfaction from therapy,<br>KT: NDI↓ post-intervention<br>ROM_{rotL}↑, ROM_{rotR}↑, post-intervention and 3-month follow-up, ROM_{flex}↑3-month follow-up, 2/14 velocity module post-intervention, 9/14 velocity module 3-month follow-up, GPE↑ post-intervention, good satisfaction from therapy, VAS↔, TSK↔, step test ↑<br>**Between-groups analysis**<br>KTVR was not superior to the KT training<br>GPE↑: KTRV>KT 3-month follow-up<br>ROM_{flex}: KTVR >KT post-intervention<br>ROM_{rotR}: KTVR< KT post-intervention<br>Peak velocity (R and L): KTVR< KT 3-month follow-up<br>Mean velocity (R): KTVR< KT 3-month follow-up

Sarig Bahat et al., 2018 [30]	**N**	**Y**	**Y**	**Y**	**N**	**N**	**Y**	**N**	**Y**	**Y**	**Y**	**7/10**	**Good**	**Within-group analysis**<br>VR group: NDI↓, Vmean↑, Vpeak↑, VAS↓, EQ5D↑, TSK↔, NVP↔, ROM↔, TTP%↑ (except TTP$_E$%), Accuracy error↓ (except accuracy error$_E$)<br>Laser group: NDI↓, Vmean↑ (except RR), Vpeak↑ (except LR), VAS↓, TSK↔, NVP$_E$↑, NVP$_{RR}$↑, ROM↔, TTP%↑ (except TTP$_E$% & TTP$_{RR}$%), Accuracy error↔<br>CG: NDI↔, Vmean ↔, Vpeak↔, VAS↔, EQ5D↔, TSK↔, NVP↔, ROM↔, TTP↔, Accuracy error↔<br>**Between-groups analysis**<br>NDI, Vmean, Vpeak (except LR), TTP$_F$% & TTP$_{LR}$%, Accuracy error (F & RR): VR>CG<br>VAS, Vmean (except RR), Vpeak (except F and RR), EQ5D, Accuracy error$_F$: VR> Laser<br>There were no group GPE differences.<br>The number of home exercise sessions was significantly higher for the laser group (mean 18.29 ± 8.63 or 4.5 times per week) compared to the VR group (14.36 ± 5.78 or 3.5 times per week).
Tejera et al., 2020 [31]	**Y**	**Y**	**N**	**Y**	**N**	**N**	**N**	**Y**	**Y**	**Y**	**Y**	**6/10**	**Good**	**Within-group analysis**<br>VR: VAS↓, ROM ↔, NDI↓, PPT↑, TSK↓, PCS↓, TS↔, CPM↔, FABQ↓<br>CG: VAS↓, ROM ↔, NDI↓, PPT↑, TSK↓, PCS↓, TS↔, CPM↔, FABQ↓<br>**Between-groups analysis**<br>VAS, ROM, NDI, PCS, PPTs, CPM, TS: VR=Control<br>TSK: VR>Control (VR Group had better improvement in 3 months follow-up)
Nambi et al., 2020	**Y**	**Y**	**N**	**Y**	**N**	**N**	**N**	**Y**	**Y**	**Y**	**Y**	**6/10**	**Good**	VR and IKT groups: VAS ↓ after 4 weeks and 6 months, Kinesiophobia ↓ after 4 weeks and 6 months

[33]														Blood serum stress hormones: subjects with cLBP exhibited a slightly greater improvement when using VR compared to IKT
Garcia et al., 2021 [27]	**Y**	**Y**	**Y**	**Y**	**N**	**N**	**N**	**Y**	**Y**	**Y**	**N**	**6/10**	**Good**	Pain intensity↓, pain-related interference in activity↓, mood↑, and stress post-treatment↓, user satisfaction, patient's Global Impression of Change, PROMIS physical function: EaseVRx > Sham 2D VR<br>Treatment engagement, usability, sleep disturbance: EaseVRx = Sham 2D VR<br>Neither group evidenced a significant change in opioid treatment, pain catastrophizing, pain self-efficacy, and pain acceptance.
Cetin et al., 2022 [28]	**Y**	**Y**	**N**	**Y**	**N**	**N**	**N**	**N**	**Y**	**Y**	**Y**	**5/10**	**Fair**	**Within-group analysis**<br>VR: ROM↑, JPSEs↑, PPTs↑, muscle strength↑, muscle endurance↑, ProFitMap-Neck↓, some parameters of SF-36↑<br>MC: ROM↑ (except lateral flexions), some PPTs↑, muscle strength↑, ProFitMap-Neck↓, some parameters of SF-36↑<br>**Between-groups analysis**<br>VAS, muscle strength, muscle endurance, HADS, SF-36, ProFitMap-Neck, PPT_{UT}, PPT_{TA}: VR=MC<br>$PPT_{C1/C2}$ & $PPT_{C5/C6}$, JPSEs: VR>MC<br>ProFitMap-Neck (functional limitation index) VR>MC

CG= Control Group, cLBP= Chronic Low Back Pain, CPM=Conditioned Pain Modulation, E= Extension, F= Flexion, EQ5D= EuroQol 5 Dimension, FABQ=Fear-Avoidance Beliefs Questionnaire, GPE= Global perceived effect, HADS= Hospital Anxiety and Depression Scale, IKT=Isokinetic Training, JPSE= Joint Position Sense Error, KT= Kinematic Training, KTVR=Kinematic Training plus Virtual Reality, LR= Left Rotation, N=No, NVP = Number of velocity peaks, PCS= Pain Catastrophizing Scale, PPT = Pressure Pain Threshold, Pro-Fit-Map neck=Profile Fitness Mapping Neck Questionnaire, PROMIS= Physical Function and Sleep Disturbance, RR= Right Rotation, ROM= Range of Motion, SF-36= 36-item short form survey, SMG= Sensorimotor Group, TS= Temporal Summation, TSK=Tampa Scale for Kinesiophobia, TTP% =Time to peak velocity percentage, TTP% =Time to peak velocity percentage, TTP_E%= Time to peak velocity percentage for Extension, TTP_F%= Time to peak velocity percentage for Flexion, TTP_{RR}%= Time to peak velocity percentage for Extension for Left Rotation, TTP_{RR}%= Time to peak velocity

percentage for Extension for Right Rotation, Vmean-= mean velocity, Vpeak= peak velocity, VAS= Visual Analogue Scale, VR= Virtual Reality, VRT= Virtual Reality Training, VRG=Virtual Reality Group, Y= Yes
